# Spatial effects in polymer chemistry

**DOI:** 10.3762/bjoc.13.198

**Published:** 2017-09-27

**Authors:** Helmut Ritter

**Affiliations:** 1 Institute of Organic Chemistry and Macromolecular Chemistry, Heinrich-Heine-University, Universitätsstraße 1, 40225 Düsseldorf, Germany

**Keywords:** polymer, spatial effects

Our modern life is no longer conceivable without macromolecular materials. Important developments in materials science, for example in the field of medical technology, electronic communication, transport and energy technology, became only possible thanks to the extensive development in the field of polymer chemistry. Although a large number of polymeric materials have already taken their place in the market, there is still a great need to develop novel materials for specific purposes and corresponding practical applications. Consequently, the synthesis and modification of macromolecules remain high priorities in scientific research.

Neighboring group effects and cross over space effects play a crucial role for the chain growth and chemical conversion of polymers in many cases. Among the spatial effects are H-bonds, van der Waals interactions, ionic forces, dipolar interactions, self-ordering effects and steric influences.

Through IR-spectroscopic studies we have recently found that the carbonyl group of, e.g., poly(acrylates) show different IR signals when positioned side by side. If, for example, they are separated by styryl units, these carbonyl signals are clearly shifted. Such effects also play an important role in the reactivity of, e.g., ester-side groups.

For example, the tacticity of a polymeric chain is a result of spatial interactions between the active growing chain end and the free monomer or a monomer–metal complex. Moreover, the preferred head-to-tail chain growth of vinyl monomers can be a result of such spatial effects. The spatial arrangement of polymer chains in the solid phase is not only influenced by external forces, for example, during extrusion, but is often also a result of chain mobility and strong intermolecular interactions. It should also be mentioned that the solubility of polymer chains is a spatial interplay between the solvent molecules and the polymer chains. Here, the LCST effects fit into the dynamic, space-spreading strength model.

I am convinced that this systematic approach provides the insights to allow a targeted and rapid development of new materials and methods. Findings concerned with spatial effects can be further explored by modern spectroscopic methods and model tests. The latter can often be carried out in a result-oriented manner, which accelerates the gain of knowledge.

Some of these points are emphasized in the present Thematic Series and may offer different perspectives for developments – right now and in the near future.

Helmut Ritter

Düsseldorf, July 2017

